# High and Ultra-High Coercive Materials in Spring-Exchange Systems—Review, Simulations and Perspective

**DOI:** 10.3390/ma15196506

**Published:** 2022-09-20

**Authors:** Artur Chrobak

**Affiliations:** Institute of Physics, University of Silesia in Katowice, 75-Pułku Piechoty 1A, 41-500 Chorzów, Poland; artur.chrobak@us.edu.pl

**Keywords:** permanent magnets, hard magnetic materials, Monte Carlo magnetic simulations, spring-exchange magnets

## Abstract

The paper refers to the spring-exchange magnetic systems containing magnetically soft and hard phases. This work consists of two parts. The first part is a brief review of hard magnetic materials, with special attention paid to ultra-high coercive compounds, as well as selected spring-exchange systems. The second part is a theoretical discussion based on the Monte Carlo micromagnetic simulations about the possible enhancement of the hard magnetic properties of systems composed of magnetically soft, as well as high and ultra-high coercive, phases. As shown, the analyzed systems reveal the potential for improving the |*BH*|_max_ parameter, filling the gap between conventional and Nd-based permanent magnets. Moreover, the carried-out simulations indicate the advantages and limitations of the spring-exchange composites, which could lead to a reduction in rare earth elements in permanent magnet applications.

## 1. Introduction

Magnetic materials have a special meaning in the progress of modern applications. Taking into account the continuous technological development of such fields as the automotive, electronics, and energy industries, the search for new magnetic materials with unique characteristics is a challenge and goal of many research teams worldwide. It may be asserted that there would be no development in many fields of technology (for example, the so-called pro-ecological technologies) without new, more efficient magnetic materials. Exemplary, modern soft magnetic materials reduce energy losses in power network transformers and electric motors. In this case, an increase in efficiency even by a few percent means a huge energy saving, thereby reducing the burden on the natural environment. In another case, hard magnetic materials are widely used as permanent magnets, among others, in generators, electric drives or various types of sensors, actuators, etc. In both cases, the most important aspects of the magnets are the price and availability of elements and, above all, the ability to optimize their properties for specific applications. 

In the field of hard magnetic materials, scientific efforts have been made to discover new magnetic materials without or with a reduced content of rare earth (RE) elements [1,2,3,4,5,6,7]. In this context, it is important to analyze some magnetic properties including physical and economic quantities. In the case of permanent magnets, the most important goal is to achieve as large a magnetic energy product |*BH*|_max_ as possible. This parameter indicates the strength of the stray magnetic field outside the material and shows how “strong” a permanent magnet made of this material can be. Until now, the best permanent magnets are based on the RE-Fe-B and RE-Co phases, for which the |*BH*|_max_ parameter is in the range of approximately 150 kJ/m^3^ (e.g., SmCo_5_) to 450 kJ/m^3^ (e.g., Nd_2_Fe_14_B). The main disadvantage of this group of magnets is their high price and the restricted availability of their RE elements. On the contrary, one can indicate the so-called classical permanent magnets such as barium ferrites (|*BH*|_max_ ≈ 30 kJ/m^3^) or the ALNICO group of alloys (|*BH*|_max_ ≈ 80 kJ/m^3^). Thus, improving the parameters of RE-free hard magnets remains one of the most important directions of research [8,9]. Even a cursory review of the literature shows that the following systems are considered in this field: (i) nanostructured magnets, (ii) thin layers, (iii) tetragonal compounds of transition metals (FeCo, FePt, and FeNi), and (iv) magnetic composites containing a magnetically hard phase (high coercivity and relatively low saturation) and soft phase (very low coercivity and high magnetic saturation). The latter seems to be the most promising. 

Indeed, in some conditions, it is possible to combine these two features to obtain a composite with a high saturation and a simultaneous high coercivity. This requires direct exchange coupling between the hard and soft phases, which can force their coherent reverse magnetization (a change of magnetization starting from full saturation in an arbitrary direction to the opposite one). In an ideal case, such a composite should have coercivity of the hard phase and magnetic remanence as saturation of the entire system (as in the so-called spring-exchange systems). In practice, it has turned out that the reverse magnetization process is more complex than the ideal scenario. There is a competition between different kinds of energies that “work” toward the pro- and anti-improvement of the |*BH*|_max_ parameter. Magnetic anisotropy energy (crystalline, surface, shape, strain, etc.) acts in the direction of the desired magnetic hardening, while magnetostatic energy (interaction of the magnetic moments with an external magnetic field) causes a reduction in the coercive field of the composite. This means that we cannot add any amount of the soft phase because this increases the magnetostatic energy and makes the reverse magnetization process “easier”. A commonly observed effect is a decrease in coercivity with an increase in magnetic saturation. In other words, the increase in saturation “costs” coercivity. Therefore, the magnetic hardness of a hard magnetic component is the most important in such composites. Quite recently (in the last decade), one has been able to obtain materials with ultra-high coercivity, with values exceeding 4 T and up to approximately 9 T at room temperature [10,11]. Until now, these materials have not had a clear spectrum of application. However, ultra-high coercive systems seem to be perfect candidates for spring-exchange magnetic composites, with a reduced contribution of RE elements.

The aim of this paper is to provide a brief review of magnetic materials with high and ultra-high coercive fields and to analyze the magnetic characteristics of the selected spring-exchange systems containing these materials. It should be noted that the role of ultra-high coercivity, which is a relatively new feature of magnetic magnets, has led to new possibilities for magnet applications. Additionally, some selected RE-free compounds and systems with Nd-/Sm-based phases are considered. In particular, the reduction in the coercivity and the improvement of the |*BH*|_max_ parameter for systems with reduced contents of RE elements (even down to zero) are discussed in detail. Particular attention is paid to computer simulations as research tools in studying the magnetic properties of complex composite materials, indicating the perspective and limits of spring-exchange systems.

## 2. Brief Review of High and Ultra-High Coercive Magnetic Materials

**Figure 1:**
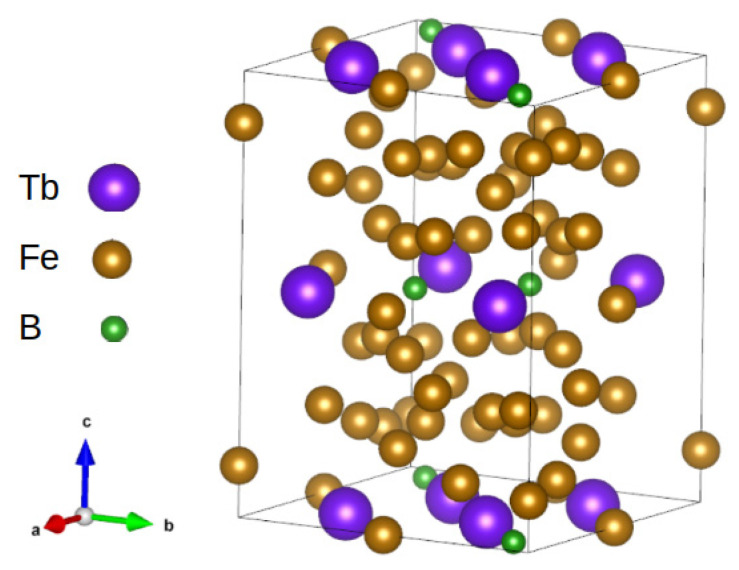
Crystal structure of Tb_2_Fe_14_B.

**RE-Fe phases**: The group of RE_2_Fe_14_B compounds is known to be a basis for high-energy permanent magnets. The hard magnetic properties of this compound’s family are related to a crystal structure and a type of RE element. A non-symmetric crystal structure with a simultaneous strong spin-orbit coupling can be a source of the so-called magnetocrystalline anisotropy simply described by the uniaxial anisotropy constant *K*_1_ [12]. The RE_2_Fe_14_B compound reveals a tetragonal structure (P4_2_/mnm), as shown in Figure 1, for the Tb_2_Fe_14_B compound. When analyzing magnetic properties, a magnetic moment of RE and a kind of magnetic order of RE-Fe attributed to specific exchange interactions are important. It is well-known that RE-Fe magnetic moments are coupled ferromagnetically for the so-called light RE elements (with an atomic weight of less than Gd), and the resulting magnetization is a superposition of both elements. In the case of heavy RE elements (i.e., an atomic weight of higher than Gd), the magnetic order between the RE element and Fe is antiferromagnetic, resulting in a relatively low magnetization. The so-called easy magnetization axis is placed along the *c* direction.

Unfortunately, a higher spin–orbit coupling occurs in the case of heavy RE elements (e.g., Dy, Tb, or Ho). Therefore, we observe either a high magnetic saturation or an ultra-high coercivity. It is possible to combine high saturation with ultra-high coercivity with alloys containing heavy and light RE elements, reaching a coercive field of approximately 3 T for (Nd/Dy)-Fe-B alloys [13,14,15]. Another important factor is the technology that can significantly influence the final properties and application possibilities. In this context, it is necessary to distinguish between RE-Fe systems as solid alloys and powders. Alloys can be obtained by different techniques such as arc melting, suction casting, or melt-spinning, while powders can be compacted by sintering or bonding methods. Moreover, hard magnetic properties can be enhanced by processing under an external magnetic field and/or mechanical pressure. The Tb-Fe-B and Dy-Fe-B systems are the most promising in the field of ultra-high coercive materials. Higher values of coercivity of approximately 9 T were obtained for Tb_2_Fe_14_B fabricated by a melt-spinning technique [11], and more than 7 T was obtained in the case of (Fe_80_Nb_6_B_14_)_0.88_Tb_0.12_ bulk alloys fabricated by the vacuum suction casting method [10]. The replacement of Tb by Dy also leads to the appearance of an ultra-high coercivity of approximately 5.5 T [14].

**Figure 2:**
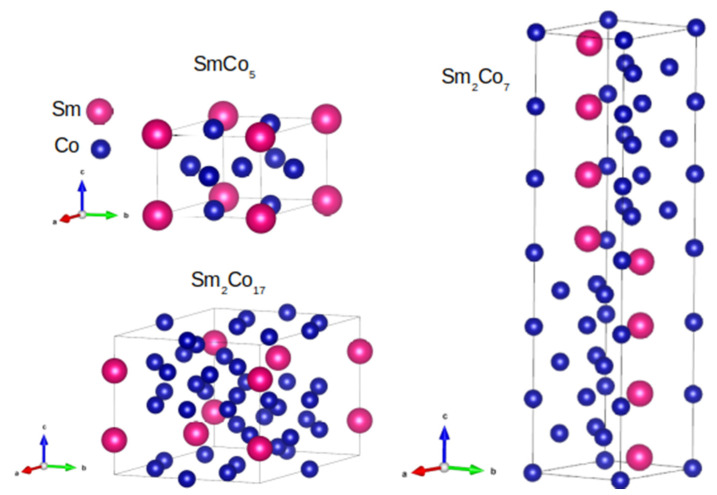
Crystal structures of the selected Sm-Co compounds.

**RE-Co phases**: The hard magnetic RE-Fe-B compounds reveal relatively low Curie temperatures of approximately 380 °C, which limits the application of these materials at high-temperatures. The substitution of Fe by Co in many compounds and alloys leads to a remarkable increase in the Curie point. For example, well-known Sm-Co systems [16,17,18,19] such as SmCo_5_ (hexagonal structure, P6/mmm), Sm_2_Co_7_ (hexagonal structure, P6/mmc), and Sm_2_Co_17_ (rhombohedral structure, P6/mmc) are important. The adequate crystal structures of these systems are presented in Figure 2. The magnetic order of the presented Sm-Co structures is ferromagnetic, with the easy magnetization axis placed along the *c* direction. Generally, conventional (bulk and powder) Sm-Co compounds are good high-temperature permanent magnets, but they have a lower energy product |*BH*|_max_ than Nd-Fe-B and a similar coercivity (not exceeding 1.2 T). Focusing on materials with ultra-high coercivity, the Sm_2_Co_7_ compound was obtained by applying the so-called hot deformation, for which the coercive field reached the value of approximately 5 T [18]. 

**Figure 3:**
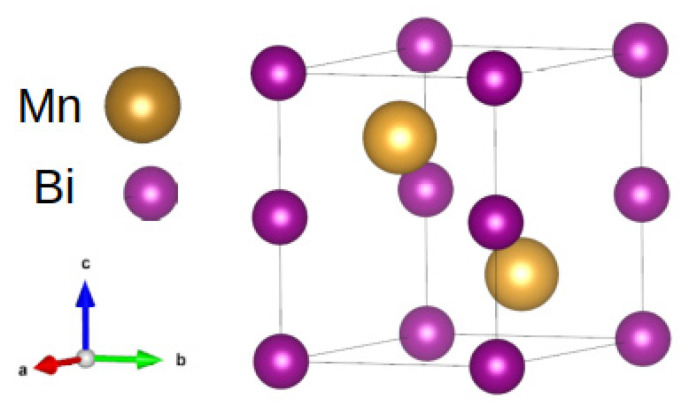
Crystal structure of the low-temperature MnBi phase.

**RE-free systems**: In recent years, much effort has been devoted to finding RE-free materials that can be used in permanent magnet applications. The main research areas include: (i) new compounds based on transition metals with non-cubic structures, (ii) the induction of magnetic anisotropy by structural distortion, and (iii) the application of surface magnetic anisotropy in nano and thin layer systems. Focusing on materials with high coercivity, the Mn-Bi, FePt, and FeCo systems are noteworthy. For such systems, in some cases, the values of the coercive field can exceed 3 T at room temperature. Further, the Mn-Bi system is interesting and promising [20,21,22], especially the low-temperature phase (LTP), which reveals high magnetocrystalline anisotropy at room temperature. The crystal structure of such a compound (MnBi, sometimes referred to as α-MnBi in the literature) is hexagonal (NiAs-type), as shown in Figure 3. At whole temperature range, the magnetic order of Mn magnetic moments is ferromagnetic. At lower temperatures of below 90 K, a spin reorientation occurs and the direction of magnetic moments changes from the *c* axis to the *ab* plane. The preparation of MnBi is difficult due to two factors: (i) the peritectic reaction between Mn and Bi, which causes Mn to solidify and segregate from the Mn-Bi liquid, and (ii) the eutectic reaction between Bi and MnBi at 535 K (any process at higher temperatures will cause phase decomposition). The MnBi bulks, powders, and thin layers are known in the literature. However, the theoretical |*BH*|_max_ value of 159 kJ/m^3^ has not been achieved due to the mentioned difficulty in obtaining the pure MnBi phase.

For example, in the case of bulk samples,|*BH*|_max_ = 58 kJ/m^3^; for powders, |*BH*|_max_ = 58 kJ/m^3^; and for a 1 µm layer, |*BH*|_max_ = 135 kJ/m^3^. Some enhancement of the energy product valued at approximately 200 kJ/m^3^ was observed in a bilayer system of MnBi (20 nm of thickness) and Co (3 nm of thickness). A higher coercivity of approximately 2.7 T was reported for thin layers prepared by the pulse laser deposition method [21].

**Figure 4:**
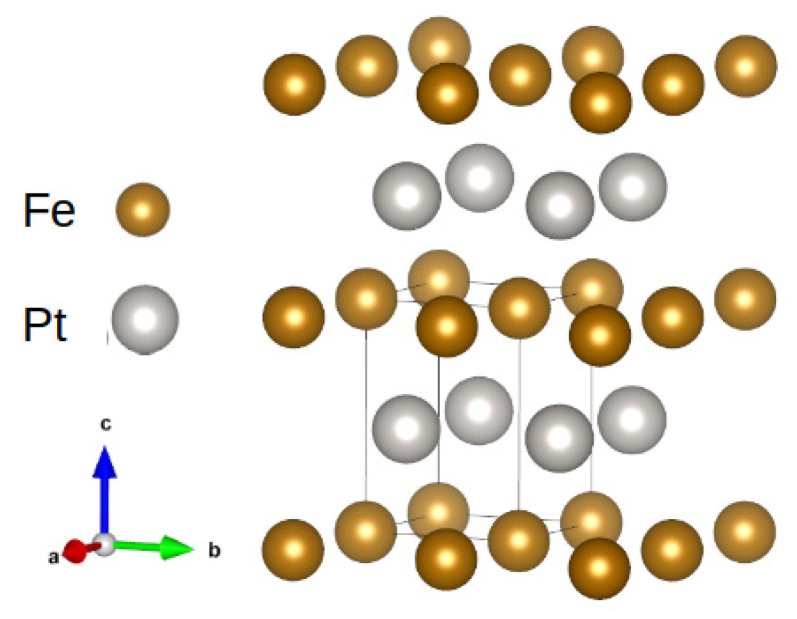
Crystal structure of the L1_0_ FePt phase.

In recent decades, L1_0_-based compounds have been intensively studied. The L1_0_ crystal structure is a tetragonal distortion of an fcc structure in which some sites in a specific face are occupied by one type of atom [23]. An example of L1_0_ FePt is shown in Figure 4. This arrangement of atoms is responsible for the appearance of high magnetocrystalline anisotropy due to a decrease in crystal symmetry. 

The FePt, FeCo, and FeNi phases are ferromagnets, and they show a high potential for ultra-high coercive materials. The FeNi compound is interesting from an economic point of view, but its bulk form has appeared (so far) only in natural iron meteorites [24,25,26,27,28]. The formation of the desired L1_0_ structure requires an extremely low cooling rate of approximately 1–5 K per million years. Nevertheless, such a structure can be obtained in thin layers and powder systems. The nitridation of A1-FeNi powders with ammonia gas is a promising technology. Finally, it is currently possible to obtain magnets with a coercivity of approximately 1.8 T [27]. 

A similar difficulty exists in the preparation of the L1_0_-FeCo phase, which has revealed a higher magnetic saturation and theoretical |*BH*|_max_ value of approximately 1000 kJ/m^3^ [29,30]. Until now, the bulk form of pure L1_0_-FeCo has not been obtained, but two strategies for the technology used can be found. The first involves the application of external forces (such as stress between two epitaxial layers), while the second involves causing the desired lattice distortion by internal forces through adding a third element. Moreover, a high coercive phase of FeCo was obtained using electron beam lithography (nanopatterning), but the value of the coercive field slightly exceeded 0.5 T [31]. 

In the discussed group of materials, the highest achieved coercivity was observed for the L1_0_-FePt compound [32,33,34,35], but its economic value is under discussion due to the high price of Pt. The technology of this phase is relatively easy and can be realized: (i) by high-temperature thermal annealing (leading to the transformation of fcc to an L1_0_ lattice structure), (ii) by chemical reactions (such as the decomposition of platinum acetylacetonate and iron pentacarbonyl in the presence of oleic acid and oleylamine, and the co-reduction in metal salts), (iii) by pyrolysis [34], and (iv) by sputter deposition. In the last case, the coercivity of the Fe_45_Pt_45_Ag_10_-C film reached the value of 4.85 T, which places this system in the family of ultra-high coercive materials [32]. The selected magnetic parameters of the reviewed compounds are listed in Table 1 [36,37,38].

Generally, the coercive field is a result of the competition between the magnetostatic and anisotropy energies. Magnetostatic energy is the main driving force for the reverse magnetization process, while anisotropy energy acts as an energy barrier for the reversal of atomic magnetic moments. Therefore, ultra-high coercivity can be expected in materials with a low saturation magnetization (or the magnetic polarization *J*_s_) and, at the same time, a high anisotropy constant *K*. In fact, the highest values of the coercive field have been observed for Tb_2_Fe_14_B, Dy_2_Fe_14_B, Sm_2_Co_7_, and FePt. 

**Figure 5:**
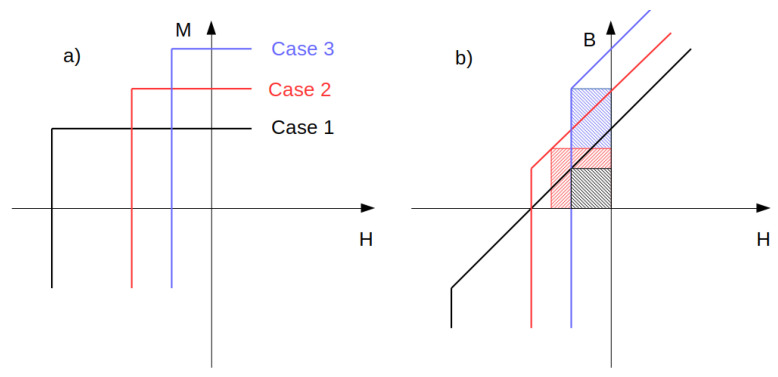
Impact of increasing the soft magnetic components on the *M*(*H*) and *B*(*H*) characteristics (see the text). The areas of crossed rectangles indicate the |*BH*|_max_ parameters. (**a**) The *M*(*H*) curve (**b**) The *B*(*H*) curve.

**Spring-exchange systems**: As shown, high coercivity is combined with relatively low magnetic saturation and remanence. The idea for an improvement is a combination of hard (usually RE-based) and soft (usually RE-free, e.g., Fe) magnetic materials, where the desired increase in remanence can occur through direct exchange coupling between these phases. The increasing contribution of the soft magnetic phase always leads to an increase in saturation and, unfortunately, to a decrease in the coercivity of the composite. This effect can be easily explained. In the situation when the magnetostatic energy (enlarged by the soft phase) surpasses the anisotropy energy, the reverse magnetization process occurs in a lower magnetic field. A question may arise regarding the optimal composition of hard and soft phases regarding the maximum value of the |*BH*|_max_ parameter. Figure 5 shows the impact of increasing the amount of the soft phase on the theoretical |*BH*|_max_ for the three cases marked by an increase in the content of the soft phase. One can notice that a coercivity of µ_0_*H*_c_ higher than the remanence polarization *J*_r_ (*J* = µ_0_*M*) does not cause any increase in the |*BH*|_max_ parameter, as seen in case 1 in Figure 5. From this simple analysis, one can state that a coercivity of higher than approximately 2.5 T does not bring any achievements for permanent magnet applications because the saturation polarization does not reach these values (the higher value is for FeCo, 2.3 T). This effect constitutes a limit for the soft phase content and, simultaneously, the possibility of the desired improvement of the |*BH*|_max_ parameter through the interaction between the ultra-high coercive and magnetically soft phases.

There are real spring-exchange magnets such as sintered powder composites [39,40,41], core-shell nanoparticles [42], and multi-layer systems [43,44] that are known in the literature. In all cases, the expected decrease in coercivity with an increase in soft component was observed. However, the spring-exchange mechanism allowed for enhancing the |*BH*|_max_ parameter by 80% for FePt-Co core-shell nanoparticles [42], 30% for a SmCo-Co thin layer system [44], and 25% for a α-FeCo/Pr_2_Fe_14_B nanocomposite [39]. Moreover, progress has been reported for hard–soft ferrite systems, with the |*BH*|_max_ reaching a value of nearly 30 kJ/m^3^ [40]. One can state that there is a limit for increasing the |*BH*|_max_ that depends on a specific system. For this reason, the study of the magnetization processes of the spring-exchange systems containing different magnetic phases, as well as different compositions, is of great importance.

## 3. Results of Simulations and Discussion

Firstly, ultra-high magnetic coercivity is, itself, interesting. Materials revealing such a property can be used in any applications where extreme resistance to an external magnetic field is required. One can mention sensors, micro-electro-mechanical systems (MEMS), or special data recording media. Nevertheless, given the applications of permanent magnets (electric motors, generators, and many “green technologies”), special attention should be paid to the spring-exchange systems utilizing ultra-high coercivity. Helpful research tools include micromagnetic simulations, which allow for the theoretical study of the magnetization processes of the considered systems. In this paper, so-called reverse magnetization curves were simulated by the Monte Carlo method. From such dependencies, the coercive field, remanence, and the |*BH*|_max_ parameter can be determined. As materials for further consideration, some selected spring-exchange systems composed of ultra-high coercive compounds with changing volume fractions of the magnetically soft components have been taken into account. Additionally, simulations for other interesting systems, according to the presented review, were also carried out. The disorder-based MC algorithm (developed by our team) was used as a computation method [45,46]. It is important that the algorithm allows for simulating the magnetization processes of systems containing magnetically different phases, and it has successfully been used in the study of similar objects [47]. For a detailed description, see [45]. In addition, the simulation parameters used in this paper are the same as those in [47].

**Figure 6:**
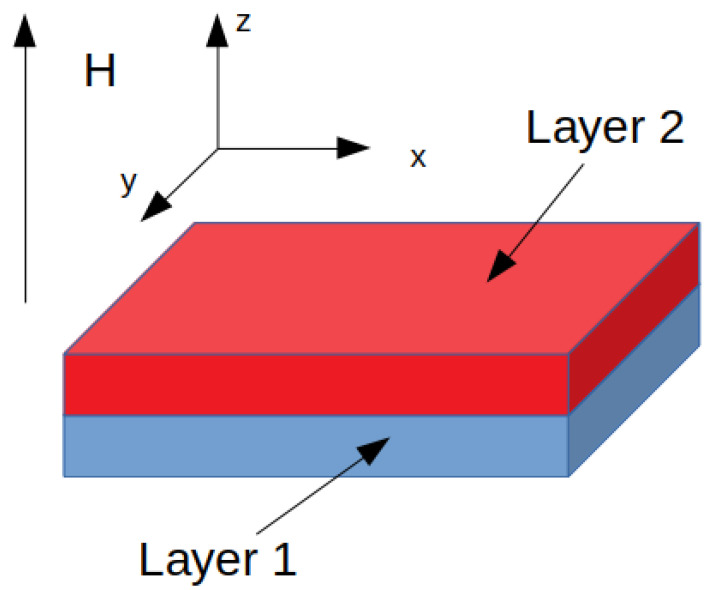
Image of the simulated bilayer spring-exchange system.

The considered systems (3D in space) consist of 40 x 40 x 40 nodes, equally spaced at a distance of 1 nm. Some nodes are attributed to the hard magnetic phase and the others to the soft, as shown in Figure 6. The bilayer system was chosen in order to simulate strong exchange coupling between the magnetically hard (blue in Figure 6) and soft (red in Figure 6) phases. The layers are infinite in the x-y plane (with periodic boundary conditions of 40 x 40 x 40 nodes per system) and a different size in the *z-*direction in order to set different phase compositions (a percentage ratio of “layer 2” to “layer 1”) in the range of 0–85%. In particular, the following systems (hard layer 1–soft layer 2) were considered: Tb_2_Fe_14_B–Fe, Nd_2_Fe_14_B–Fe, SmCo_5_-Fe, FePt–Fe, FeCo–Fe, and MnBi–Fe. Additionally, the Tb_2_Fe_14_B–SmCo_5_, Tb_2_Fe_14_B–FePt, Nd_2_Fe_14_B–SmCo_5_, and Nd_2_Fe_14_B–FePt bilayer systems were analyzed.

According to the Hamiltonian used in our method [45,47], the simulations require establishing the values of the spin *S*, anisotropy constant *K*, and exchange integral parameter *J,* assuming that the nodes are equally spaced at a distance of 1 nm. The spin values were calculated with the assumption that the saturation magnetization of the simulated system is equal to the mass magnetization of the real compound. The parameters *K* and *J* were determined to obtain the real values of the coercive fields and the Curie temperatures. Table 2 presents all the considered compounds and elements used as magnetic layers, including their parameters. 

**Figure 7:**
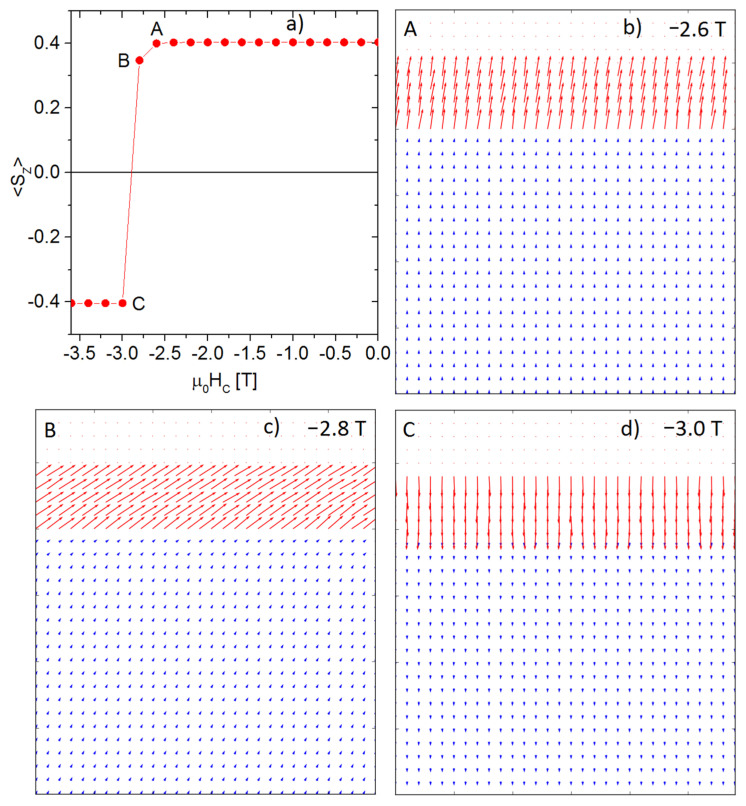
Reverse magnetization curve and spin configurations for the Tb_2_Fe_14_B-Fe (20% Fe) system near the coercivity point. (**a**) reverse magnetization curve (**b**) spin configuration for the point A (**c**) spin configuration for the point B (**d**) spin configuration for the point C.

The main results of the carried-out simulations are the reverse magnetization curves, from which it is possible to determine remanence, coercivity, and the |*BH*|_max_ parameter. An example, the curve for Tb_2_Fe_14_B-Fe (20% Fe) is shown in Figure 7a. The rapid reverse magnetization at the coercive field is related to the assumed simplification that the external field is placed parallel to the direction of magnetic anisotropy. Figure 7b,c depicts the spin configurations for the different external fields around coercivity.

**Figure 8:**
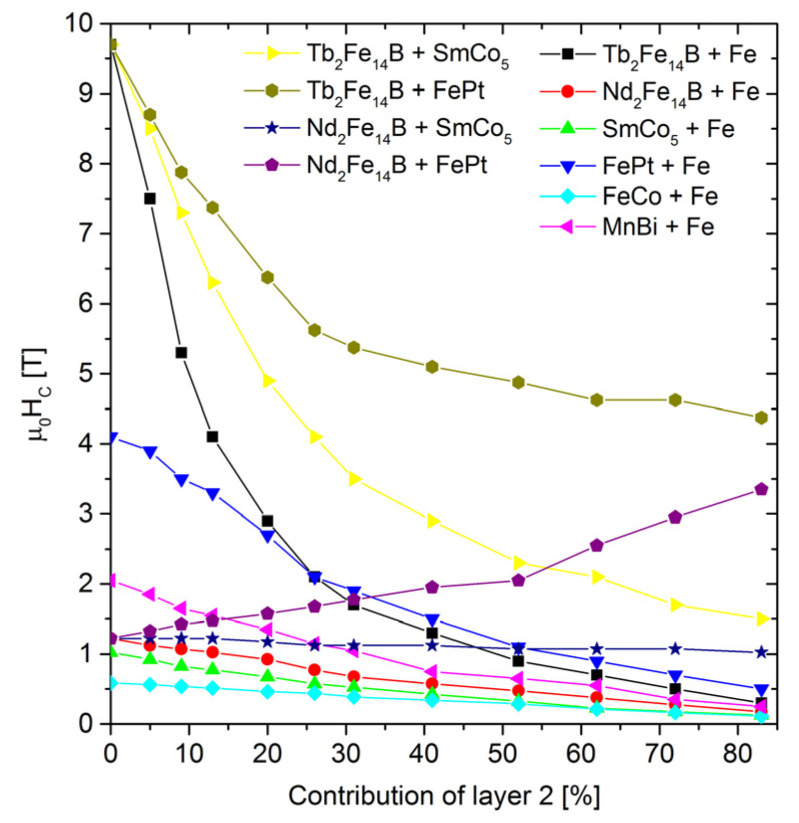
Saturation polarization as a function of the “layer 2” content.

**Figure 9:**
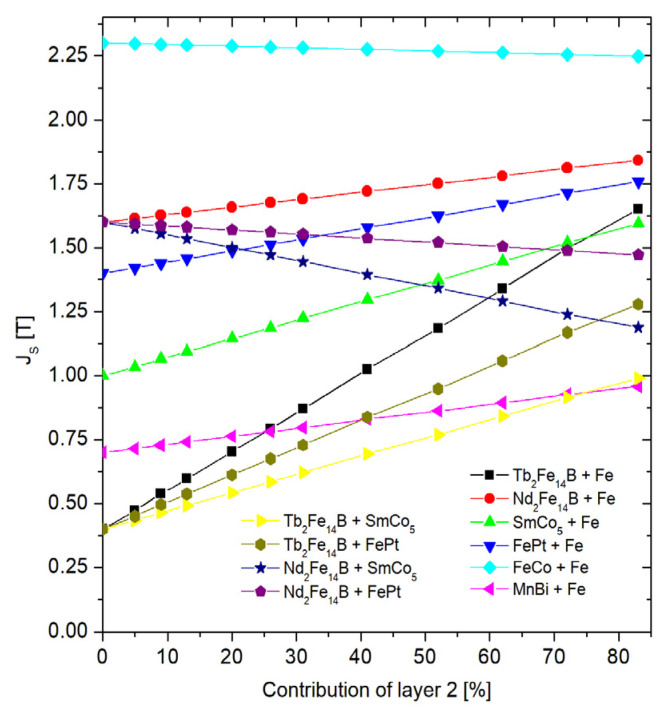
Saturation polarization in a function of the “layer 2” content.

**Figure 10:**
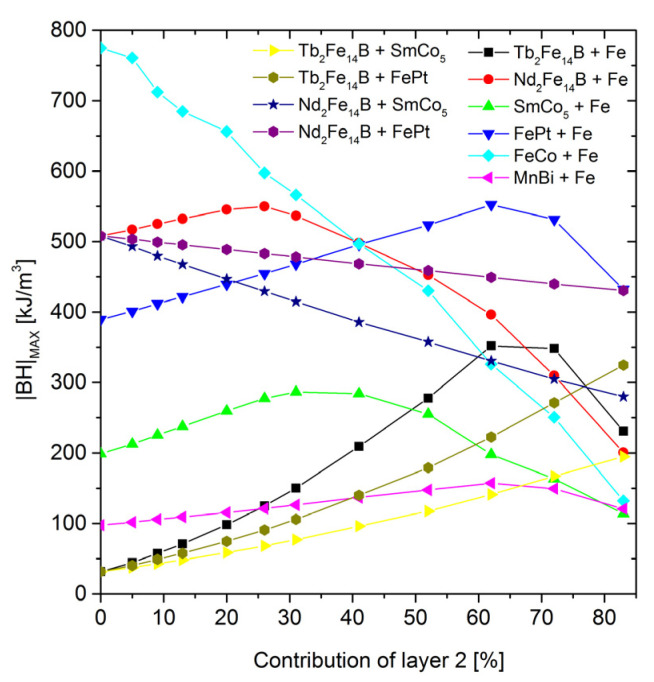
The |*BH*|_max_ parameter as a function of the “layer 2” content.

**Figure 11:**
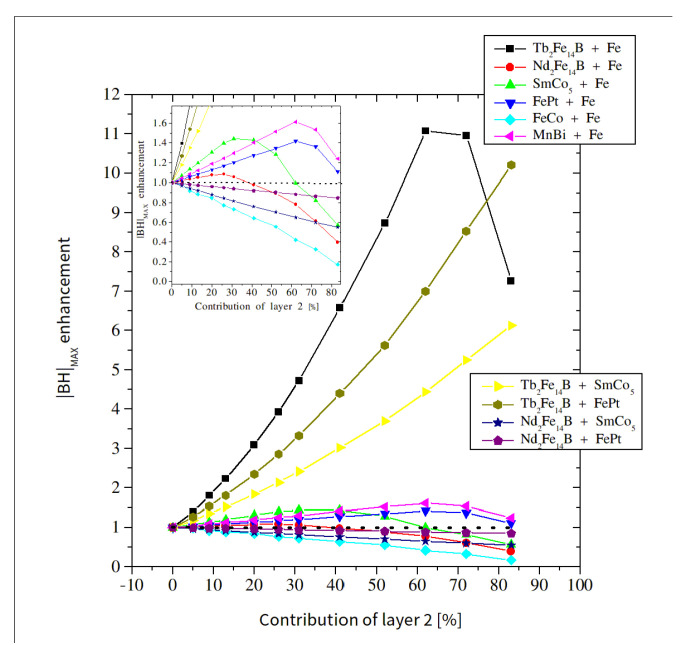
Relative change of the |*BH*|_max_ parameter as a function of the “layer 2” content.

Figure 8 shows a collection of coercivities in a function of the composite composition for all the examined cases. A general tendency to decrease the *H*_c_ with an increasing contribution of the magnetically soft (or softer) components can be observed. The strongest coercivity decay is observed for the system of Tb_2_Fe_14_B-Fe. However, for 60% of Fe, the coercive field still exceeds 1 T. The dependence of the *H*_c_ vs. the soft phase content is rather exponential, ranging from coercivity of the hard phase to coercivity of the soft phase. Obviously, the strongest coercivity decay is observed and expected with the largest difference in the *H*_c_ between these phases. The increase in the content of the soft component causes a linear change in the saturation magnetization and, consequently, the magnetic remanence, as shown in Figure 9. The remanence and coercivity are parameters that strongly influence the |*BH*|_max_. In the case of the analyzed systems, the increase in the contribution of the soft phase with a high saturation level causes an increase in the residual magnetization and, simultaneously, a decrease in the coercive field. The first tendency leads to an increase in the |*BH*|_max_, while the second acts in the opposite direction. Finally, the optimal composition of the composite should be observed. Figure 10 and Figure 11 show the dependence of the content “layer 2” (mainly soft phase) on the |*BH*|_max_ and the relative change of this parameter (in relation to the pure hard phase), respectively. In some cases, a maximum |*BH*|_max_ occurs, and the corresponding values of the |*BH*|_max_, enhancement of the |*BH*|_max_, and the “layer 2” contents are shown in Table 3. 

The maximum is not observed for the FeCo-Fe composite due to the decrease in magnetic saturation with the increasing Fe contribution. If the composite contains two hard magnetic phases, then the |*BH*|_max_ parameter varies monotonically. Excluding the economic aspect, some systems without or with a reduced RE content will fill the gap between Nd-based and classical permanent magnets, i.e., between 50 and 400 kJ/m^3^. The special importance of ultra-high coercive phases as a part of the spring-exchange composite should be emphasized because the values of the |*BH*|_max_ in the gap range occur for a much smaller RE contribution. In the case of Tb_2_Fe_14_B-Fe, FePt-Fe, and FeCo-Fe composites with 80% Fe, these are still good permanent magnets. It is interesting to compare the predicted and experimental enhancement of the |*BH*|_max_ for the studied cases. One can indicate a relatively good agreement for the MnBi-Fe system (simulations revealed an enhancement of approximately 1.6 times, i.e., from 99 kJ/m^3^ to 160 kJ/m^3^). As reported in [48], for the MnBi-Fe multilayer system, the |*BH*|_max_ varied from 100 kJ/m^3^ to 140 kJ/m^3^, which gives a satisfactory compatibility with the results of the presented simulations. Another experimentally studied thin layer system is SmCo-Co [44], for which a 30% increase in the |*BH*|_max_ was reported. The simulations indicated that this value in the level of 47%, accounting for simplifications of the model, can be a confirmation of the correctness of the modeling assumptions. The obtained result of the |*BH*|_max_ enhancement for the Nd-Fe-B/Fe system (theoretically, approximately 10%) seems to be surprising. In this case, the coercivity decay is “faster” than the increase in remanence, which is the main reason for such an effect. In fact, there is no information in the literature reporting significant progress in applying the spring-exchange mechanism to this type of system. For other examined cases, it is hard to perform such a comparison due to difficulties in obtaining a pure L1_0_ phase, as well as in assuming the theoretical values of the anisotropy constants in the simulations. The Tb-Fe-B/Fe system has not been experimentally tested. However, in case of Tb-Fe-B/Y-Fe-B alloys, the |*BH*|_max_ enhancement reached a value of approximately 300% [49].

**Figure 12:**
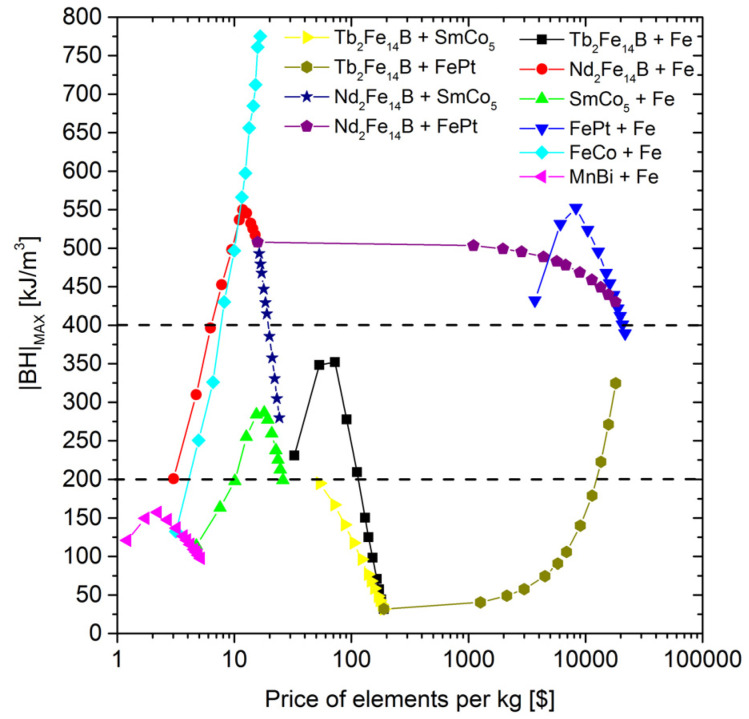
The |*BH*|_max_ parameter as a function of the estimated price of the elements.

**Figure 13:**
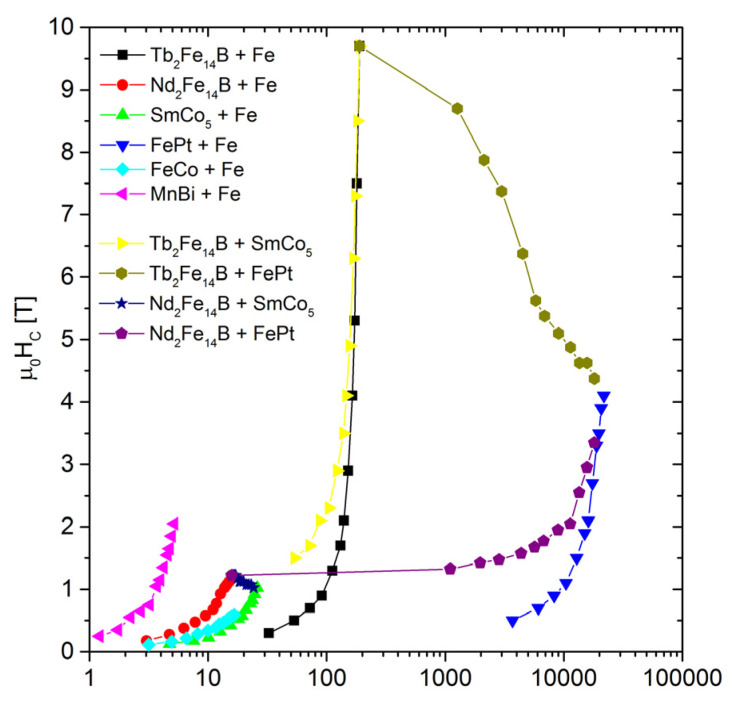
Coercive field as a function of the estimated price of the elements.

Unfortunately, the mentioned composites are expensive due to the high price of Tb and, especially, Pt. The data received should be collected as a function of the average price of the elements. Figure 12 shows such a function for the |*BH*|_max_ parameter. One can see that only Nd_2_Fe_14_B-Fe, SmCo_5_-Fe, MnBi-Fe, and FeCo-Fe fill the gap, with prices of below 10 USD/kg. Figure 13 presents the cost aspect of coercivity. As shown, ultra-high coercivity can be obtained only for systems with Tb_2_Fe_14_B and FePt. The cost of a composite with a coercivity of more than 3T is over 100 USD/kg (for Tb_2_Fe_14_B) and 1000 USD/kg (for FePt).

## 4. Concluding Remarks

Ultra-high magnetic coercivity (arbitrary, higher than 3 T) is not a common feature of magnetic materials. Such a property requires an extremely high magnetic anisotropy and, simultaneously, a low saturation magnetization. This combination can be found in the case of RE-Fe-B (RE = Tb, Dy, and Ho), where the magnetic moments of RE and Fe are antiferromagnetically coupled. The highest value of the coercive field, i.e., approximately 9 T, is observed for Tb_2_Fe_14_B-based systems for melt-spun ribbons, and values of more than 7 T were observed for bulks produced by the vacuum suction technique. The main disadvantages of the Tb-Fe compound are its low remanence and high price. Therefore, much effort is spent on finding new RE-free materials. The most promising are tertragonal distortions of Fe-Ni, Fe-Co, and Fe-Pt systems that occur in the L1_0_ phase. The highest coercivity (less than 5 T) was observed for the FePt L1_0_ phase, but the economic value of this compound is under discussion.

As was shown, a coercivity that exceeds the remanence polarization does not lead to an improvement of the |*BH*|_max_. This means that a coercive field at the level of approximately 2.5 T is sufficient for the mentioned applications. Coming back to the |*BH*|_max_ parameter, of interest and surprise is the gap between conventional (e.g., ALNICO, |*BH*|_max_ < 50 kJ/m^3^) and Nd-based permanent magnets (|*BH*|_max_ ≈ 400 kJ/m^3^). Filling this gap with RE-free materials is the main aim of the current progress in research on hard magnetic materials. The spring-exchange composite containing hard and soft magnetic phases seems to be one such material. The idea is to develop a suitable composition in which the hard phase is the source of the magnetic anisotropy, while the soft phase contributes to an increase in the remanence. Unfortunately, the resulting coercive field decreases with an increasing amount of soft phase. In permanent magnet applications, ultra-high coercive materials appear to be very useful, and such composites can contain much more of the magnetically soft phase. For example, the carried-out simulations reveal that the composites Tb_2_Fe_14_B-Fe and FePt-Fe, with a 25% addition of Fe, have a coercive field at the level of 2.5 T. Another aspect is the optimal phase composition regarding the maximum |*BH*|_max_ parameter, as well as filling the gap between RE-based and RE-free permanent magnets. As shown, the most effective improvement of the |*BH*|_max_ occurs in the case of ultra-high coercive phases (e.g., approximately 12 times for Tb_2_Fe_14_B-Fe with a maximum of approximately 350 kJ/m^3^). On the contrary, for the Nd_2_Fe_14_B-Fe composite, the addition of 25% Fe causes an increase in the |*BH*|_max_ parameter by approximately 10%. Considering the price of the composite, MnBi-Fe and FeCo-Fe composites are interesting. For MnBi-Fe, the |*BH*|_max_ parameter does not exceed 150 kJ/m^3^, while in the case of FeCo-Fe, the addition of 60–70% Fe results in this parameter being at the level of 300 kJ/m^3^.

Possible applications for ultra-high coercive materials are not obvious. However, in some cases in which extreme resistance from external magnetic fields is required, this feature can be helpful. As a future idea, one can mention a source of a magnetic field in micro-sensors, micro-actuators (MEMS devices), or data recording media. At present, the main application areas seem to be spring-exchange systems in which either improving the hard magnetic properties or decreasing the RE elements content can be expected.

The carried-out analysis is partially speculative. However, the system design and the chosen parameters for simulations ensure, at least, its theoretical correctness. The possibility of obtaining real composites with effective-enough exchange coupling is under question. Nevertheless, the presented results can expand existing knowledge about the advantages and limitations of the applications of ultra-high coercive materials in new spring-exchange composites.

## Figures and Tables

**Table 1:** Selected magnetic parameters (*K*_1_ is the anisotropy constant, *H*_A_ is the anisotropy field, *H*_c_ is the coercive field, and *J*_s_ is the remanence polarization) for the high and ultra-high coercive phases.

Compound	*K*_1_ (MJ/m^3^)	µ_0_*H*_A_ (T)	µ_0_*H*_C_ (T)	*J*_s_ (T)	References
Nd_2_Fe_14_B	4.9	15.1	1.2	1.6	[9,36]
Tb_2_Fe_14_B	6.16	35.2	>7 T (bulk) 9 T (ribbon)	0.56	[10,11,36]
Dy_2_Fe_14_B	4.24	31.4	5.5	0.565	[15,36]
Ho_2_Fe_14_B	2.4	25.1		0.642	[36]
SmCo_5_	13	30.5	1.1–1.2	1.14	[9,36]
Sm_2_Co_7_	4.5	>20	5.07	1.02	[16,18,19]
Sm_2_Co_17_	4.2	5.4	1.1–1.2	1.25	[9,19,37]
MnBi	1.6	5	1.3–2	0.91	[20]
FeNi	1.1–1.3	1.44	0.07	1.47	[27]
FeCo	10 (theory) 2.1 (layer)		0.6	2.3	[31,38]
FePt	6.6		4.85	1.4	[3,6,32,34]

**Table 2:** Magnetic parameters for the selected compounds used in the simulations.

Compound/Element	*S*	*K* (eV)	*J* (eV)
Tb_2_Fe_14_B	0.23	1.3 × 10^−4^	0.17
Nd_2_Fe_14_B	0.93	6.5 × 10^−5^	9.80 × 10^−3^
SmCo_5_	0.64	3.6 × 10^−5^	9.80 × 10^−3^
FePt	0.84	2.0 × 10^−4^	1.50 × 10^−2^
FeCo	1.13	3.8 × 10^−5^	1.46 × 10^−2^
MnBi	0.76	8.7 × 10^−5^	1.56 × 10^−2^
Fe	1.1	0	1.23 × 10^−2^

**Table 3:** The |*BH*|_max_, parameters, |*BH*|_max_ enhancements, coercive field *H*_c_s, and the “layer 2” contents at the optimal points.

System	|*BH*|_max_ (kJ/m^3^)	|*BH*|_max_ Enhancement (%)	µ_0_*H*_c_ (T) at Optimum	Layer 2 Content (%)
Tb_2_Fe_14_B-Fe	380	1200	0.62	67
Nd_2_Fe_14_B-Fe	552	10	0.81	26
SmCo_5_-Fe	295	47	0.47	36
FePt-Fe	560	43	0.84	66
MnBi-Fe	160	64	0.45	66
